# Guides for facilitating the implementation and evaluation of social prescribing: lessons from the “Access to Resources in the Community” model

**DOI:** 10.24095/hpcdp.44.9.07

**Published:** 2024-09

**Authors:** Kiran Saluja, Simone Dahrouge

**Affiliations:** 1 Bruyre Research Institute, Ottawa, Ontario, Canada; 2 Department of Family Medicine, University of Ottawa, Ottawa, Ontario, Canada

**Keywords:** social prescribing, patient navigation, toolkit, navigator training

## Abstract

Social prescribing (SP) embodies a comprehensive approach to addressing the social determinants of health. Access to Resources in the Community (ARC) is an innovative SP program offering bilingual services that involves a single point of entry for health and social needs and introduces practice changes to assist primary care providers in engaging patients, along with a nonclinical lay navigator who supports patients in accessing relevant community resources. The ARC team has created a SP toolkit offering practical guidance for setting up, implementing, monitoring the progress of and evaluating SP programs. The four ARC guides can be easily customized for application in diverse practice and research settings.

**Highlights**

- We developed a set of guides for use in “Access to Resources in the Community” (ARC), one of the first social prescribing (SP) programs established and evaluated in Ontario, Canada.
- The four guides are: (1) PC Practice Set Up, (2) Navigator Training, (3) Navigation Processes and (4) Evaluation. The guides provide practical guidance for establishing, conducting and monitoring progress, and evaluating SP programs; they form the basis of a toolkit we created to support organizations and researchers in establishing and evaluating SP programs.
- The guides and toolkit are currently being adopted for the ARC/211- Ontario program that we are cocreating through a nonprofit research partnership with Community Connections, an innovative hub of 211 Ontario in Collingwood. The program will help generate evidence on the feasibility, effectiveness, impact on health inequities and cost-effectiveness of adapting and scaling up SP programs in Canada.

## Introduction

The social determinants of health have a significant impact on the health of individuals.^1^ Social prescribing (SP) consists of the identification of patients with unmet needs related to these determinants, commonly in primary care (PC), and the provision of support to help them access the needed resources. The structure of SP varies considerably across settings. In some programs, changes are introduced to the PC practice, such as establishing practice champions and referral mechanisms, to facilitate the identification and engagement of such individuals,^2^,^3^ although many studies describing these programs do not mention any changes to practice.^4^,^5^

In some cases, the support offered to help individuals access resources may be as simple as sign-posting (providing information and/or promotional material) at the PC practice, which has less impact^6^,^7^ than the more common structure in which a trained individual, often called a link worker, provides navigation services.^4^,^8^,^9^ With some exceptions,10 the link worker is an individual outside the PC practice who supports practices within a defined region.^2^,^4^,^11^ The training and role of the link worker are often not well described;^12^,^13^ in some studies, their role is principally described as that of identifying and connecting the individual to the service,^4^ while in others it involves more intensive support. Some link workers’ functions include a structured approach to identifying access barriers and helping individuals overcome them; the co-creation of personalized plans;8 providing various levels of emotional support; advocacy; and forming strategies to build empowerment and self-efficacy.^2^,^8^,^14^

Some SP programs limit the target population to specific sociodemographic groups^12^,^15^,^16^ or to individuals with specific needs,^9^,^17^-^19^ while others target a broad population.^4^ Studies have shown SP to have varying degrees of success, which is likely because a broad range of approaches are used, with different target populations and vast variability in the outcome measures.^3^,^4^,^10^,^20^ There is evidence that more intensive support, continued patient engagement and structured referral processes are more likely to produce benefits.^2^,^17^,^21^

## Access to Resources in the Community (ARC) SP model

Over the past decade, SP has been widely adopted across the United Kingdom and is rapidly expanding internationally.^22^,^23^ Initiatives to promote SP are relatively new in Canada.^2^,^24^ In partnership with patients, providers and health planners, our team developed ARC, an innovative, PC-based SP program offering bilingual services to improve equitable access to health and social resources. The ARC approach involves a single point of entry for health and social needs, introduces practice changes to assist PC providers to engage their patients in self-care for these needs, and provides the services of a nonclinical lay navigator who supports patients to access the appropriate community resources. The ARC SP model was demonstrated to be feasible and acceptable across different PC practice models in Ontario, Canada.^2^

We subsequently conducted a randomized controlled trial to compare the ARC navigation services to the existing online navigation services provided by 211 Ontario, a free, multilingual web and telephone information and referral service for health and social resources that is available around the clock in Ontario. In that trial, PC practices applied SP as usual, but patients were randomized to either the ARC navigation service or the 211 Ontario navigation system. We assessed patient and provider experience, access to needed resources and impact on health services in the two arms. These results are in preparation for publication. 

## ARC social prescribing guides and toolkit

There is a dearth of information and resources relating to practice changes and the training required for the link worker. The tools and guides available to support the implementation and practice of SP were mostly developed in the UK,^25^-^27^ and more recently from the Alliance for Healthier Communities in Ontario.^28^ The ARC team developed a set of guides for use in the ARC research program^29^ that can provide practical guidance for establishing, conducting, monitoring the progress of and evaluating SP programs. These guides are the basis of the toolkit (https://www.arcnavigatorproject.com/sp-toolkit)^30^ we created to support organizations and researchers in establishing SP programs; the four guides are: PC Practice Set Up, Navigator Training, Navigation Processes, and Evaluation. 

***Guide 1: PC Practice Set Up***

The ARC team established simple processes for implementing SP in primary care that can readily be integrated without disrupting the practice workflow. This guide includes presentations on SP for recruitment and practice orientation to review study procedures; recommendations for practice changes to adopt social prescribing; and examples of the tools used.

***Guide 2: Navigator Training***

The navigator’s role is broad. Navigators must establish a trusting relationship with the patients, elicit information about their social context and anticipated access barriers, understand their priorities and preferences, and help build the individual’s self-efficacy. They offer informational, instrumental and emotional support to help patients overcome barriers and successfully access the needed resources. The ARC team developed a learner-centred, theoretically grounded, competency-based training program for individuals without a clinical background to acquire the competencies to carry out their role.^31^ The training involves a total of 25 hours of self-paced education sessions, covering a set of 13 training modules supplemented with face-to-face workshops, and covers the need for ongoing mentorship from experienced navigators or program managers. Each module contains study material such as PowerPoint presentations, video recordings, handouts, peer-reviewed articles and additional learning resources. 

***Guide 3: Navigation Processes***

In addition to providing patients with the support required to achieve access, the navigator also helps ensure the continuity of information across sectors by providing feedback to the PC provider about their patient’s progress and resources accessed. This guide provides a step-by-step description of the ARC navigation processes and the corresponding tools that support navigators in their role, facilitate their work and help ensure fidelity to the established processes.

***Guide 4: Evaluation***

Ongoing monitoring of SP programs, especially at the earlier stages, is necessary to identify and mitigate issues relating to fidelity, processes in place, and other factors that can compromise the success of the program. The evaluation of SP programs allows the program administrators to assess whether the initiative has achieved its intended objectives. While these are often specific to each initiative, they will also contain common elements. The ARC SP evaluation guide provides some insight and tools from our work that may be adapted for use in other SP programs. That guide covers (1) rapid cycle evaluations, to assess the impact of SP on PC practice functioning; (2) patient surveys, to assess access and patient experience; and (3) provider surveys, to assess providers’ level of satisfaction with various components of the SP program and their perception of the impact of SP on the health and well-being of their patients. 

## Scaling up SP: the ARC/211-Ontario SP program

The ARC team and the Collingwood Community Connection (CC) team, a regional initiative of 211 Ontario that has been piloting a SP program, have partnered to co-develop, implement and test a comprehensive SP model that builds on the two teams’ assets and experience. The ARC/211-Ontario model will incorporate elements of the ARC approach that support practice engagement and delivering patient-centred, longitudinal services required for more socially complex individuals, and will leverage the CC’s approach for regional SP programs, existing resources and technological innovations to enhance the structure and efficiency of the service delivery and facilitate the navigator’s work.

## Acknowledgements

We thank Darene Toal-Sullivan and Carolynn Warnet for their contribution in developing the ARC-SP training guides and toolkit. We also thank Ontario 211 and Community Connection for their collaboration with the ARC program in their role as a community navigation delivery system. 

## Conflicts of interest

The authors declare no conflicts of interest.

## Authors’ contributions and statement

SD: conceptualization, writing—review and editing.

KS: writing—original draft.

The content and views expressed in this article are those of the authors and do not necessarily reflect those of the Government of Canada.
